# Supplementary material for: Which SDM Model, CLIMEX vs. MaxEnt, Best Forecasts Aeolesthes sarta Distribution at a Global Scale under Climate Change Scenarios?
Source: Insects. 2024 May 1;15(5):324. doi: 10.3390/insects15050324 (PMC11121915; doi:10.3390/insects15050324)
Supplement: Supplementary file 1 [file insects-15-00324-s001.zip › insects-2984924-SI.pdf]

Fitting of CLIMEX parameters for *A. sarta*

Aelosthes sarta Biological Data for CLIMEX model

Due to its preference for dry, cold weather, *A. sarta* is most often found in temperate zones (Hayat 2022); thus, the Temperate template included with CLIMEX software was used to create a "species parameter file." Physiological tolerance thresholds for *A. sarta* were used to calibrate the model. The existing occurrence data of *A. sarta* was used to determine the species climatic requirements. Parameter values for temperature, moisture, and diapause index (Table 3) were defined using inferred data from the published literature associated with developmental threshold temperatures, moisture, and diapause (Hayat 2022; Khan et al. 2013; Farashiani et al. 2000). By repeatedly running the parameter file with different parameter values, the stress index values were fine-tuned until the predicted climate suitability patterns were consistent with *A. sarta's* observed distribution.

Temperature Index

In the beginning model was run using temperature values as DV0 = 11°C, DV1 = 20°C, DV1 = 35°C and DV4 = 40°C (Vanhanen et al. 2008; Ahmad et al. 1977). However, these values did not predict *A. sarta's* occurrence in northern India, China, and Afghanistan. Hence, DV0, DV1, and DV2 were adjusted to 10°C, 15°C and 37°C (Hayat et al. 2023) to include positive sites in Northern India, China, and Afghanistan and were finalized to forecast the potential distribution globally. The population day degree (PDD) value for A. sarta was set to be 700 (Vanhanen et al. 2008; Hayat et al. 2023), as it completes its lifecycle in almost two years (Hayat 2022; Khan et al. 2013; Farashiani et al. 2000; Ahmad et al. 1977).

Diapause Index

For *A. sarta*, Diapause Development Days (DPD) were set at 90days (Hayat et al. 2023), as literature reported that *A. sarta* diapause duration is approximately 90 days (Hayat 2022; Khan et al. 2013; Farashiani et al. 2000). Other diapause parameters such as Diapause induction day length (DPD0), Diapause induction temperature (DPT0), Diapause termination temperature (DPT1), and Summer or winter Diapause (DPSW) were set at 12 days, 13°C, 10°C and 0 days respectively (Vanhanen et al. 2008; Hayat et al. 2023).

Moisture Index

Another parameter the CLIMEX model uses is the soil moisture index (MI). SM0, SM1, SM2, and SM3 (for detail names, please see Table 3) are the four additional characteristics that the CLIMEX model uses to categorize the MI further (Sutherst et al. 2007). For normal species development, SM0 was attuned to 0 (Vanhanen et al. 2008; Hayat et al. 2023). SM1 and SM2 were set 0.001 and 1.5 (Hayat et al. 2023)[41]. Following a similar practice, the value for SM3 was set at 2.5 (Vanhanen et al. 2008; Hayat et al. 2023).

2.4.4. Stress Index

Dry stress (DS), wet stress (WS), cold stress (CS), and heat stress (HS) are four environmental stress indices in the CLIMEX that depict unfavorable conditions that limit a species' population development (Sutherst et al. 2007). For *A. sarta,* only HS and CS were considered. The cold stress restricts species' development when the temperature falls below the cold stress threshold temperature (TTCS) of that species at a particular rate (THCS). In the present study, TTCS was set at 9°C as below this temperature *A. sarta* development is impeded (Ahmad et al. 1977). Following the TTCS value, the THCS value was set to -0.00001. This rate provided an appropriate fit to the observed occurrence of *A. sarta.* However, when temperatures rise above a species' heat stress temperature threshold (TTHS), the growth of that species is hindered. As *A. sarta* cannot survive above the temperature of 40°C (Ahmad et al. 1977), the TTHS value was set at 41°C. The weekly rate (THHS) was set at 0.005 (Hayat et al., 2023). The observed global distribution of *A. sarta* was well matched by these values.

References

[1] Hayat U. City longhorn beetle (Aeolesthessarta): A review of the species, its morphology, distribution, da-mage, prevention and control. Journal of Forest Science, 2022, 68, 199-212. https://doi.org/10.17221/34/2022-JFS

[2] Khan SA, Bhatia S and Tripathi N. Entomological investigation on Aeolesthessarta (Solsky), a major pest on walnut trees (Juglans regia L.) in Kashmir valley. Journal of Academia and Industrial Research 2013, 2(6), p.325.

[3] Farashiani ME, Shamohammadi D and Sadeghi SE. Biological study of Sart long horn beetle, AeolesthessartaSolsky (Coleoptera: Cerambycidae) in the laboratory. Journal of Entomological Society of Iran 2000, 20(1), 77–90.

[4] Vanhanen H, Veteli TO and Niemelä P. Potential distribution ranges in Europe for Aeolesthes sarta, Tetropiumgracilicorne and Xylotrechusaltaicus, a CLIMEX analysis. EPPO bulletin 2008, 38, 239–248. https://doi.org/10.1111/j.1365-2338.2008.01211.x

[5] Ahmad MI, Hafiz IA and Chaudhry MI. Biological studies on Aeolesthes sartus Solsky attacking poplars in Pakistan. Pakistan Journal of Forestry 1977, 27(3), 122-129.

[6] Sutherst RW, Maywald GF and Bourne AS. Including species interactions in risk assessments for global change. Global change biology 2007, 13(9), 1843-1859. http://doi.org/10.1111/j.1365-2486.2007.01396.x

[7] Hayat, U.; Akram, M.; Kour, S.; Arif, T.; Shi, J. Pest Risk Assessment of Aeolesthes sarta (Coleoptera: Cerambycidae) in Pakistan under Climate Change Scenario. Forests 2023, 14, 253. https://doi.org/10.3390/f14020253

<u>Fitting of CLIMEX parameters for *Populus alba*</u>

1. Host (*Populus alba*) Ecological Data

Populus alba is primarily found in temperate climates with moist and relatively cold weather (Pasiecznik 2022); thus, a Temperate template included with CLIMEX software was used to create a "species parameter file." Parameter values for temperature and moisture index (Table A) were determined using data inferred from the literature (Nevidomov 1994; Modir-Rahmati 1997; Pasiecznik et al. 2005; Af-shan et al. 2011; Pasiecznik 2022; Sekawin 1975; Gucker 2010). By running the parameter file with varying parameter values, we were able to fine-tune the stress index values so that the predicted climate suitability patterns were consistent with *P. alba's* observed global distribution.

2. Temperature Index

Populus alba cannot tolerate frigid temperatures, and its growth effects at low temperate such as -5°C (Pasiecznik 2022; Sekawin 1975), whereas temperatures higher than 35°C might be problematic for the growth of P. alba in temperate regions (Gucker 2010; Domingo and Gordon 1974). Therefore, lower (DV0) and upper (DV3) temperature thresholds for growth, after which species stopped growing, were set to -5 and 35°C. DV0, DV1, DV2, and DV3 values used to run the model were -5°C, 4°C, 20°C and 35°C respectively.

3. Moisture Index

Populus alba is a tree species that grows well in both moist and dry soils (Rédei 1998)[53]. However, areas with moist soil conditions and moderate to heavy precipitation rates favor the growth rate of *P. alba* (Pasiecznik 2022; Sekawin 1975; Gucker 2010; Domingo and Gordon 1974). As a result, restricting low soil moisture (SM0) was set at 0.5 to enable normal species growth, and SM1 and SM2 soil moisture values were set up for optimal growth. In addition, the SM3 soil moisture upper limit was set to be 2.5.

4. Stress Index

For *P. alba*, all four stress indices, CS, HS, WS, and DS, were considered. The cold stress restricts species' development when the temperature falls below the cold stress threshold temperature (TTCS) of that species at a particular rate (THCS). Three different cold temperature values (-5°C, -15°C and -45°C) were used to run the model, at -5°C, many known distribution points were missing; at -15°C and -45°C all known distributions were covered, and no notable difference was found. Therefore, In the current study, TTCS was set at -15°C as *P. alba* growth is inhibited at extremely low temperatures (Pasiecznik 2022; Gucker 2010). A THCS value of -0.0001 was determined from the TTCS data. The same corresponds to heat stress; once

temperatures rise above a species' heat stress threshold (TTHS) at a given rate (THHS), the growth and development of that species will be impeded. In the current study, TTHS was established at 35°C because *P. alba* does not thrive above this temperature (Gucker, 2010)[51]. The weekly rate (THHS) was set at 0.005. Likewise, dry stress also affects a species' development. In the present research, SMDS was set at 0.2 as *P. alba* cannot grow well under low soil moisture conditions (Domingo and Gordon 1974). The dry stress accumulation rate (HDS) was set at -0.005. Through an algorithm of iteration, the wet stress value was mapped to the known distribution of *P. alba*. Therefore, the wet stress threshold (SMWS) was set at 2.5 with an accumulation rate (HWS) of 0.002, respectively.

Table A. Parameter values used in the CLIMEX model for *Populus alba*.

	Parameters	Code	Values
			Populus alba
**Temperature**	Limiting low temperature (°C)	DV0	-5
	Lower optimal temperature (°C)	DV1	4
	Upper optimal temperature (°C)	DV2	20
	Limiting high temperature (°C)	DV3	35
**Moisture Index**	Limiting low soil moisture	SM0	0.5
	Lower optimal soil moisture	SM1	1
	Upper optimal soil moisture	SM2	1.5
	Limiting high soil moisture	SM3	2
**Cold Stress**	CS temperature threshold (°C)	TTCS	-15
	CS temperature rate	THCS	-0.0001
**Heat Stress**	HS temperature threshold (°C)	TTHS	35
	HS temperature rate	THHS	0.005
**Wet Stress**	Wet stress threshold	SMWS	2.5
	Wet stress accumulation rate	HWS	0.002
**Dry Stress**	Dry stress threshold	SMDS	0.2
	Dry stress accumulation rate	HDS	-0.005

References

[1] Nevidomov AM. Ecophytocoenotic patterns in the distribution of poplar forests in the floodplains of southeastern European Russia. Botanicheskiĭ Zhurnal, 1994, 79(12), pp.47-58.
[2] Modir-Rahmati A. Cultivation of poplar in Iran. Holzzucht, 1997, 51(2/2), 41-43.
[3] Pasiecznik NM, Smith IM, Watson GW, Brunt AA, Ritchie B and Charles LMF. CABI/EPPO distribution maps of plant pests and plant diseases and their important role in plant quarantine. Eppo Bulletin, 2005, 35(1), 1-7.
[4] Afshan NS, Iqbal SH, Khalid AN, Niazi AR, 2011. Some additions to the uredinales of Azad Jammu and Kashmir (AJ & K), Pakistan. Pakistan Journal of Botany, 2011, 43(2), 1373-1379. http://www.pakbs.org/pjbot/PDFs/43(2)/PJB43(2)1373.pd
[5] Pasiecznik N. 'Populus alba (silver-leaf poplar)', CABI Compendium. CABI International, 2022. https://doi.org/10.1079/cabicompendium.43426
[6] Sekawin M. Genetics of Populus alba. Annales Forestales, 1975, 6(6), 159-189.
[7] Gucker CL. Populus alba and hybrids. In: Fire Effects Information System, [Online]. U.S. Department of Agriculture, Forest Service, Rocky Mountain Research Station, Fire Sciences Laboratory (Producer), 2010. Available at: https://www.fs.usda.gov/database/feis/plants/tree/popspp/all.html [accessed on 2023 March 15].
[8] Domingo IL and Gordon JC. Physiological responses of an aspen-poplar hybrid to air temperature and soil moisture. Botanical Gazette, 1974, 135(3), 184-192. https://www.journals.uchicago.edu/doi/abs/10.1086/336750
[9] Rédei K. Early evaluation of promising white poplar (Populus alba L.) clones in sandy ridges between the rivers Danube and Tisza in Hungary. Silva Lusitana, 1998, 6(1), 63-71.

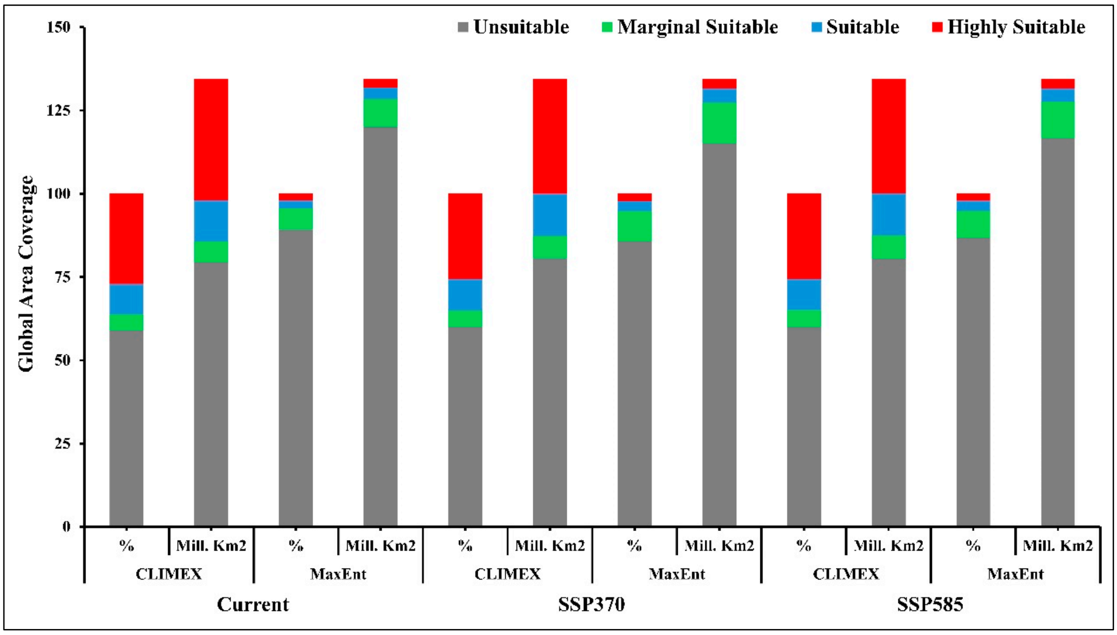

Figure S1. Percentage of the land area distribution of *Aelosthes sarta* for CLIMEX and MaxEnt models under current and future climatic conditions within the four habitat suitabil-ity ranges (Red = Highly suitable, Blue = suitable, Green = marginal suitable, and Sil-ver = unsuitable) on a global scale.
